# Structural Basis for a Neutralizing Antibody Response Elicited by a Recombinant Hantaan Virus Gn Immunogen

**DOI:** 10.1128/mBio.02531-20

**Published:** 2021-07-06

**Authors:** Ilona Rissanen, Stefanie A. Krumm, Robert Stass, Annalis Whitaker, James E. Voss, Emily A. Bruce, Sylvia Rothenberger, Stefan Kunz, Dennis R. Burton, Juha T. Huiskonen, Jason W. Botten, Thomas A. Bowden, Katie J. Doores

**Affiliations:** a Division of Structural Biology, Wellcome Centre for Human Genetics, University of Oxford, Oxford, United Kingdom; b Institute of Biotechnology and Helsinki Institute of Life Science (HiLIFE), University of Helsinki, Helsinki, Finland; c Department of Infectious Diseases, King's College London, London, United Kingdom; d Division of Immunobiology, Department of Medicine, Larner College of Medicine, University of Vermont, Burlington, Vermont, USA; e Cellular, Molecular, and Biomedical Sciences Graduate Program, University of Vermont, Burlington, Vermont, USA; f Department of Immunology and Microbiology, The Scripps Research Institute, La Jolla, California, USA; g Department of Microbiology and Molecular Genetics, Larner College of Medicine, University of Vermont, Burlington, Vermont, USA; h Institute of Microbiology, Lausanne University Hospital and University of Lausanne, Lausanne, Switzerland; i Ragon Institute of MGH, Harvard, and MIT, Cambridge, Massachusetts, USA; j Molecular and Integrative Biosciences Research Programme, Faculty of Biological and Environmental Sciences, University of Helsinki, Helsinki, Finland; McMaster University

**Keywords:** glycoprotein, hantavirus, neutralizing antibody, structure, zoonosis

## Abstract

Hantaviruses are a group of emerging pathogens capable of causing severe disease upon zoonotic transmission to humans. The mature hantavirus surface presents higher-order tetrameric assemblies of two glycoproteins, Gn and Gc, which are responsible for negotiating host cell entry and constitute key therapeutic targets. Here, we demonstrate that recombinantly derived Gn from Hantaan virus (HTNV) elicits a neutralizing antibody response (serum dilution that inhibits 50% infection [ID_50_], 1:200 to 1:850) in an animal model. Using antigen-specific B cell sorting, we isolated monoclonal antibodies (mAbs) exhibiting neutralizing and non-neutralizing activity, termed mAb HTN-Gn1 and mAb nnHTN-Gn2, respectively. Crystallographic analysis reveals that these mAbs target spatially distinct epitopes at disparate sites of the N-terminal region of the HTNV Gn ectodomain. Epitope mapping onto a model of the higher order (Gn-Gc)_4_ spike supports the immune accessibility of the mAb HTN-Gn1 epitope, a hypothesis confirmed by electron cryo-tomography of the antibody with virus-like particles. These data define natively exposed regions of the hantaviral Gn that can be targeted in immunogen design.

## INTRODUCTION

Hantaviruses chronically infect rodent, shrew, mole, and bat reservoirs worldwide (1). Zoonotic transmission of hantaviruses to humans typically occurs through exposure to aerosolized rodent excreta and can lead to severe disease. For example, Andes virus (ANDV) and Sin Nombre virus (SNV) are causative agents of hantavirus cardiopulmonary syndrome (HCPS), and Hantaan virus (HTNV), Puumala virus (PUUV), and Seoul virus (SEOV) cause hemorrhagic fever with renal syndrome (HFRS). Depending on the causative hantavirus species, case‐fatality rates for HFRS (1, 2) and HCPS (3, 4) have been reported to reach up to approximately 10% and 36%, respectively. There are currently no FDA-approved therapeutics or vaccines to treat or prevent hantavirus infection.

Hantaviruses belong to the order *Bunyavirales*, family *Hantaviridae*, and are negative-sense, single-stranded RNA viruses with a tripartite genome consisting of S (small), M (medium), and L (large) segments (1, 5). The envelope-anchored glycoproteins, Gn and Gc, are produced by enzymatic cleavage of the glycoprotein precursor (GPC) protein encoded in the M segment and are jointly responsible for orchestrating host cell entry and fusion (6–8). Hantavirus infection in humans generates a strong, long-lasting humoral antibody (Ab) response with neutralizing antibody (nAb) titers detectable >20 years post-infection (9, 10). Abs targeting the nucleoprotein (N) arise rapidly following infection, rendering them a useful biomarker for infection (11–13). However, it is the Abs that bind Gn and Gc that have neutralizing activity and provide lasting protection *in vivo* (14–17). In addition to providing protective immunity, nAbs are thought to play an important role in disease clearance during infection. For example, higher nAb titers are seen in individuals who experience milder disease (14, 18, 19), and passive transfer of convalescent-phase plasma to individuals suffering with acute HCPS may reduce fatality rates (20).

Despite the importance of nAb responses in protection against disease (15, 21–23) and disease clearance (19, 24), our understanding of the immune response against hantaviruses is limited, and there are currently no FDA-approved vaccines to prevent hantavirus-induced disease. nAbs arising following hantavirus infection in humans and from vaccination of animals target both Gn and Gc (16, 25–27). Epitope mapping studies based on neutralization escape mutants and peptide mapping have putatively identified a number of neutralizing epitopes on the hantaviral Gn and Gc (25, 27–32), and the structure of a neutralizing monoclonal antibody (mAb) in complex with a hantaviral Gc has provided insights into the molecular basis for antibody-mediated targeting of the hantaviral Gc (33).

X-ray crystallographic investigations have revealed that the N-terminal globular domain region of Gn forms a mixed α/β fold (34, 35), and the Gc forms the archetypal class II fusion fold (33, 36–38) also observed in genetically distinct flaviviruses, alphaviruses, and phleboviruses (39–42). Integration of these crystal structures with electron cryo-tomography (cryo-ET) analyses of the hantaviral surface have demonstrated that the Gn and Gc assemble to form lattices of higher-order square-like arrangements (35, 43, 44), where the globular domain of Gn protects the hydrophobic fusion loops of the Gc at a membrane-distal region of the virion surface (34–36). Recently, this assembly model was validated through determination of the crystal structure of an ANDV Gn-Gc complex (36), which revealed the interactions between Gn and Gc. Furthermore, the Gn-Gc interface has been shown to dynamically alternate between “open” and “closed” configurations, where the more loosely associated “open” conformation displays reduced fusogenic activity (45). The acidic pH that accompanies virus internalization is thought to trigger conformational rearrangements to the higher-order Gn-Gc assembly, allowing Gc-mediated merger of the host and virion envelopes (34, 46–48).

While hantaviral Gn and Gc are principal targets for the protective neutralizing antibody response (14–17), little is known about the molecular determinants that dictate the neutralizing antibody-mediated immune response arising against these glycoproteins. Here, we show that a recombinantly derived and purified Gn ectodomain fragment from HTNV, a causative agent of severe HFRS in Korea and China (1, 49, 50), elicits a nAb response (serum dilution that inhibits 50% infection [ID_50_], 1:200 to 1:850) against HTNV in an animal model. Further, we used antigen-specific B cell sorting to isolate a mAb termed HTN-Gn1, which neutralizes HTNV *in vitro* at a potency similar to that of a modestly neutralizing and protective nAb (27), and a non-neutralizing mAb termed nnHTN-Gn2. Structural characterization of HTNV Gn with the Fab fragments of these antibodies by cryo-ET and X-ray crystallography provides a structural rationale for neutralization, revealing antigenically accessible regions of Gn within the native hantaviral envelope architecture. This work demonstrates recombinant HTNV Gn as a potential target for vaccine development efforts and provides a structure-based platform for interrogating the molecular basis for the antibody-mediated targeting of the hantavirus surface.

## RESULTS

### Soluble HTNV Gn ectodomain elicits a neutralizing antibody response.

**FIG 1:**
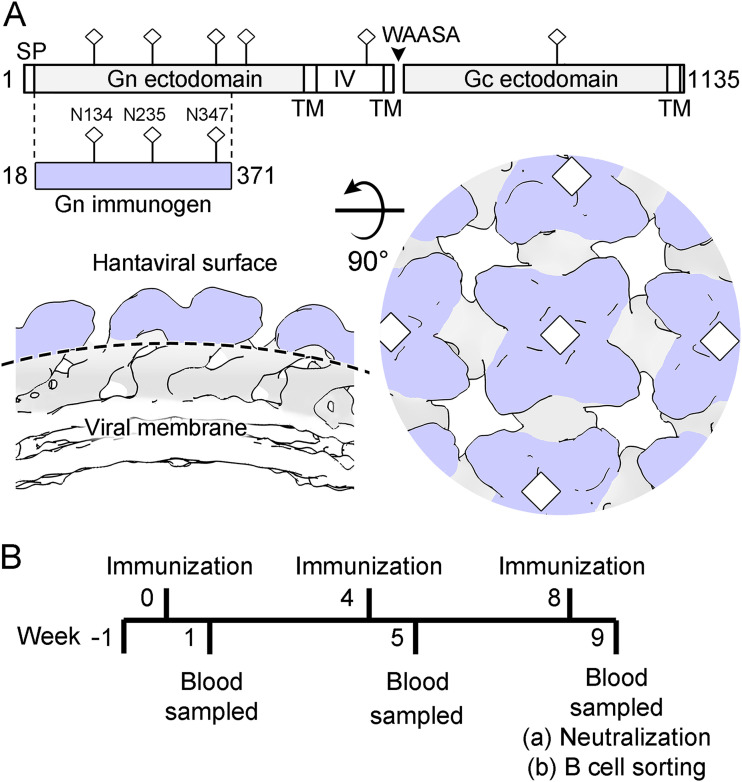
HTNV Gn immunization strategy. (A) (Upper) Schematic diagram illustrating the Gn and Gc glycoproteins encoded in the HTNV M segment. The construct of HTNV Gn (residues 18 to 371) used for immunization is highlighted and colored lilac (produced with DOG 4.0 [94]). Predicted N-linked glycosylation sites (NXT/S, where X≠P) are annotated with sticks. (Lower) Schematic diagram of the (Gn-Gc)_4_ lattice (based upon EMD-4867), as revealed by previous cryo-ET and X-ray crystallography studies (35). Although the Gc may likely impinge, the N-terminal region of the hantaviral Gn is predicted to make up the majority of the membrane-distal region (lilac) of the (Gn-Gc)_4_ lattice. (B) Timeline of rabbit immunization experiments. Rabbits were immunized with recombinant HTNV Gn and boosted at 4-week intervals. Seven days following the third immunization, HTNV Gn binding and neutralization titers were measured. mAbs were isolated through antigen-specific single B cell sorting of PBMCs (Fig. S2).

**FIG 2:**
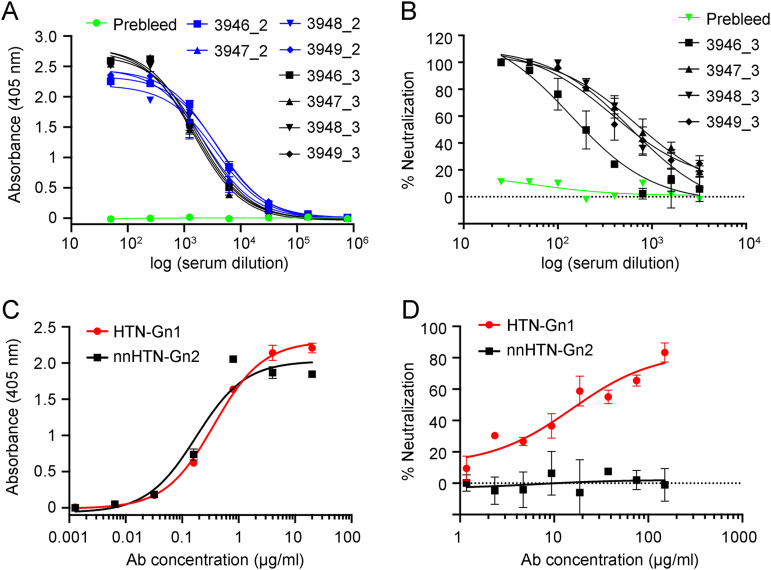
Immunization with HTNV Gn elicits a nAb response enabling isolation of neutralizing mAb HTN-Gn1 and non-neutralizing mAb nnHTN-Gn2. (A) Analysis of the IgG-specific response to HTNV Gn by ELISA in rabbit sera (rabbits 3946 to 3949) following the second (blue, indicated by _2) and third (black, indicated by _3) HTNV Gn immunizations. A prebleed serum control is shown in green. (B) Neutralization of live HTNV strain 76-118 by rabbit sera (rabbits 3946 to 3949) following the third HTNV Gn immunization. A prebleed serum control is shown in green. (C) Characterization of mAbs HTN-Gn1 and nnHTN-Gn2 binding to HTNV Gn by ELISA. (D) Neutralization of live HTNV strain 76-118 by mAbs HTN-Gn1 and nnHTN-Gn2. Despite mAb nnHTN-Gn2 exhibiting high binding to HTNV Gn, this mAb did not show neutralizing activity. Error bars represent the standard errors of the mean. In panels A and C, ELISA was carried out three times in duplicate. In panels B and D, the neutralization assay was carried out twice in duplicate. Representative graphs are shown.

Integrated crystallography and cryo-ET studies have demonstrated that the N-terminal globular region of the hantaviral Gn, a central target of the neutralizing antibody response to infection (14–17), locates to the membrane-distal tetrameric lobes of the Gn-Gc spike complex (34–36) (Fig. 1A). We sought to determine whether this region of the Gn is capable of eliciting nAbs by immunizing four New Zealand White rabbits with recombinantly derived HTNV Gn ectodomain (residues 18 to 371) (Fig. 1B). We observed an IgG binding response (half-maximal binding response ranging from 1:2,300 to 1:4,000) against HTNV Gn following the first booster immunization (Fig. 2A). Furthermore, the polyclonal antibody response neutralized live HTNV virus (Fig. 2B), with ID_50_ neutralization titers (serum dilution that inhibits 50% infection) ranging from 1:200 to 1:850. These titers are consistent with those previously observed in patients following HTNV infection (1:400 in a plaque reduction neutralization test [PRNT]) (30) and similar to those elicited following DNA M segment vaccination (PRNT ID_50_ range, 1:20 to 1:1,280), which were shown to be protective against HTNV infection in a hamster model (51).

10.1128/mBio.02531-20.2 FIG S2 Amino acid sequence comparison of isolated mAbs HTN-Gn1 (A) and nnHTN-Gn2 (B) and their corresponding germline sequence. CDRs are shown. Top germline V-gene hits were determined using the International Immunogenetics Information System (IMGT) database (M. P. Lefranc, V. Giudicelli, C. Ginestoux, J. Bodmer, W. Müller, R. Bontrop, M. Lemaitre, A. Malik, V. Barbié, and D. Chaume, Nucleic Acids Res 27:209–212, 1999, https://doi.org/10.1093/nar/27.1.209). The sequence alignment was determined by Clustal Omega (F. Sievers, A. Wilm, D. Dineen, T. J. Gibson, K. Karplus, W. Li, R. Jopez, H. McWilliam, M. Remmert, J. Söding, J. D. Thompson, and D. G. Higgins, Mol Syst Biol 7:539, 2011, https://doi.org/10.1038/msb.2011.75) and plotted with ESPRIPT (X. Robert and P. Gouet, Nucleic Acids Res 42:W320–W324, 2014, https://doi.org/10.1093/nar/gku316). Download FIG S2, PDF file, 0.4 MB. Copyright © 2021 Rissanen et al. 2021 Rissanen et al. https://creativecommons.org/licenses/by/4.0/ This content is distributed under the terms of the Creative Commons Attribution 4.0 International license.

Following the second HTNV Gn boost, we isolated HTNV Gn-specific B cells by antigen-specific single B cell sorting from peripheral blood mononuclear cells (PBMCs) (see Fig. S1 in the supplemental material). HTNV Gn-reactive B cells were sorted into individual wells, and the variable heavy and light regions of the cognate mAbs were rescued through nested PCR using rabbit gene-specific primers (52). Cloning of these regions into rabbit IgG expression vectors facilitated expression of mAbs for further characterization. Using this methodology, we were able to isolate an HTNV Gn-binding (half-maximal binding 50% effective concentration [EC_50_], 0.37 μg/ml) and modestly neutralizing mAb (half-maximal inhibitory concentration [IC_50_], 15.47 μg/ml against live HTNV), termed HTN-Gn1, and an HTNV Gn-binding (EC_50_, 0.18 μg/ml) and non-neutralizing mAb, termed nnHTN-Gn2 (Fig. 2). Sequence analysis revealed 10% divergence from germline for both the HTN-Gn1 and nnHTN-Gn2 heavy chains (V and J regions combined) and 12% and 5% divergence from germline for the HTN-Gn1 and nnHTN-Gn2 kappa chains, respectively (V and J regions combined) (Fig. S2 and Table S1).

10.1128/mBio.02531-20.1 FIG S1 Antigen-specific fluorescence-activated B cell sorting gating strategy. CD3^−^ IgM^−^ IgG^+^ HTNV Gn^+^ cells were sorted into single wells of a 96-well plate for subsequent sequence analysis. Download FIG S1, TIF file, 2.6 MB. Copyright © 2021 Rissanen et al. 2021 Rissanen et al. https://creativecommons.org/licenses/by/4.0/ This content is distributed under the terms of the Creative Commons Attribution 4.0 International license.

10.1128/mBio.02531-20.7 TABLE S1 Germline analysis of isolated mAb HTN-Gn1 and nnHTN-Gn2 sequences and their predicted germlines. Antibody germline V, D, and J genes were assigned using the International Immunogenetics Information System (IMGT) database (M. P. Lefranc, V. Giudicelli, C. Ginestoux, J. Bodmer, W. Müller, R. Bontrop, M. Lemaitre, A. Malik, V. Barbié, and D. Chaume, Nucleic Acids Res 27:209–212, 1999, https://doi.org/10.1093/nar/27.1.209). Download Table S1, DOCX file, 0.02 MB. Copyright © 2021 Rissanen et al. 2021 Rissanen et al. https://creativecommons.org/licenses/by/4.0/ This content is distributed under the terms of the Creative Commons Attribution 4.0 International license.

### Neutralizing and non-neutralizing antibodies target distinct faces of the Gn.

To elucidate the epitopes targeted by mAb HTN-Gn1 and mAb nnHTN-Gn2, we crystallized and determined the structure of HTNV Gn in complex with its cognate Fab fragments to 3.5-Å and 2.7-Å resolutions, respectively (Table S2). In both structures, Fab-associated HTNV Gn exhibits the expected mixed α/β-sandwich fold (Protein Data Bank [PDB] accession code 5OPG) (34), where an overlay of unliganded HTNV Gn with the Gn components of Fab HTN-Gn1 and nnHTN-Gn2 complexes results in matching average RMSDs (root mean square deviations) of 0.9 Å over 316 and 322 C-α atoms, respectively. Two nearly identical copies of the HTNV Gn−Fab HTN-Gn1 complex (1.2 Å RMSD over 317 aligned C-α atoms) and one copy of the HTNV Gn−Fab nnHTN-Gn2 complex were observed in the asymmetric unit of each respective crystal.

10.1128/mBio.02531-20.8 TABLE S2 Crystallographic data collection and refinement statistics for Fab HTN-Gn1−HTNV Gn and Fab nnHTN-Gn2−HTNV Gn complexes. Download Table S2, DOCX file, 0.02 MB. Copyright © 2021 Rissanen et al. 2021 Rissanen et al. https://creativecommons.org/licenses/by/4.0/ This content is distributed under the terms of the Creative Commons Attribution 4.0 International license.

**FIG 3:**
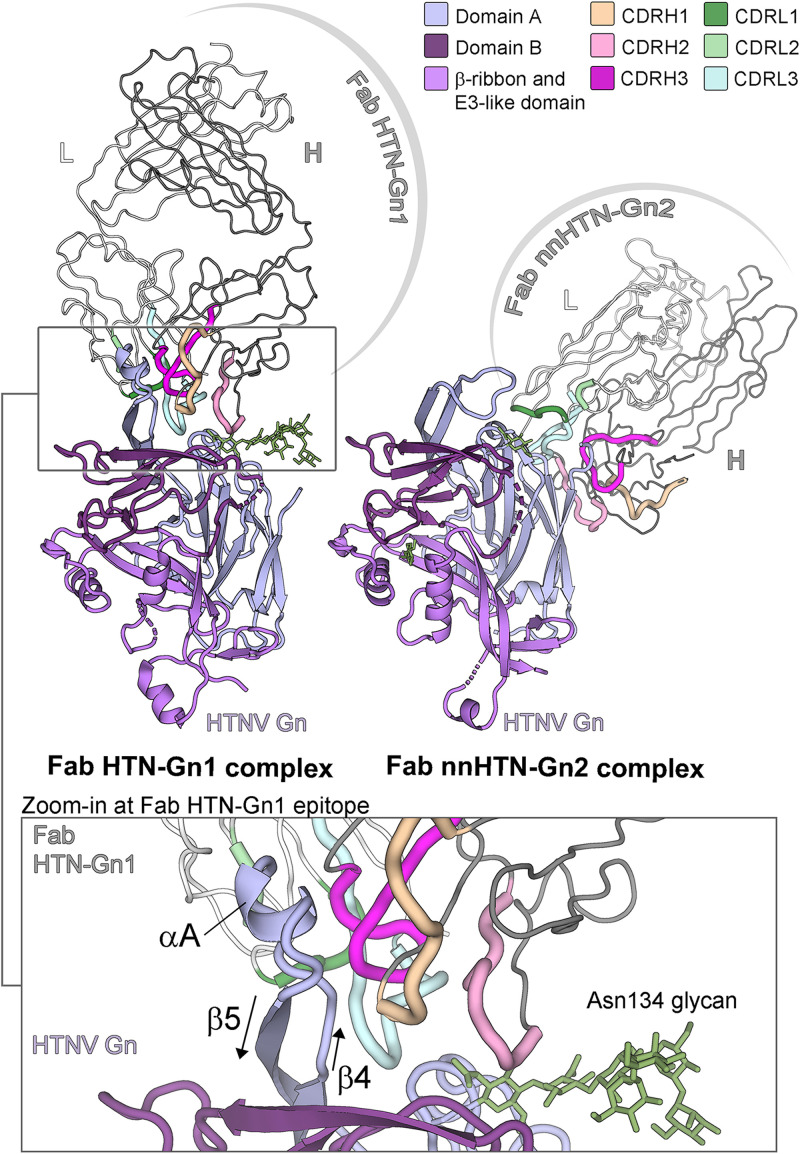
Crystal structures of Fab fragments from neutralizing mAb HTN-Gn1 and non-neutralizing mAb nnHTN-Gn2 in complex with HTNV Gn reveal that the antibodies target disparate epitopes on the HTNV Gn surface. (Upper left) Structure of the HTNV Gn−HTN-Gn1 complex. The heavy and light chains of Fab HTN-Gn1 are colored dark and light gray, respectively. The CDR loops of the Fab are colored shades of pink (heavy chain) and green (light chain), respectively, as defined in the upper right legend. HTNV Gn is colored according to domain, with domain A in light blue, domain B in dark purple, and the β-ribbon/E3-like domain in purple, as defined in the upper right legend. (Upper right) Structure of the HTNV Gn−nnHTN-Gn2 complex. Colored as described for panel A. The Fab nnHTN-Gn2 binds to domain A of HTNV Gn in an interaction that relies on the CDRs of both heavy and light chains and occludes 900 Å^2^ of surface area. (Lower) Zoom-in of the HTNV Gn−HTN-Gn1 interface. The ordered glycan extending from Asn134 (green sticks) of HTNV Gn from the Fab HTN-Gn1 complex was likely protected from endoglycosidase F_1_ during sample preparation and is stabilized by neighboring crystal contacts. Detailed representations of the interactions at the antibody-antigen interfaces are provided in Fig. S3 and S4 in the supplemental material.

Structural overlay analysis reveals that the two Fabs bind to distinct faces of HTNV Gn (Fig. 3), where Fab nnHTN-Gn2 recognizes an epitope comprised of β-sheets β1, β8, and β9 of the Gn and Fab HTN-Gn1 targets residues in the loop that links HTNV Gn strands β4 and β5 (loop_β4-β5_, residues 82 to 96) (Fig. 3; Fig. S3 and S4). Interestingly, the conformation of loop_β4-β5_ is distinct from that observed in the previously reported structure of HTNV Gn, where loop_β4-β5_ mediates contacts between HTNV Gn tetramers observed in the crystal (34). Indeed, Fab HTN-Gn1 seems to stabilize loop_β4-β5_ in a helical conformation (Fig. 3), and there is no evidence for the formation of higher-order Gn oligomers.

10.1128/mBio.02531-20.3 FIG S3 Key interactions at the Fab HTN-Gn1**−**HTNV Gn complex interface. Paratope and epitope residues involved in hydrogen bonding (dashed black lines) and hydrophobic interactions (dashed blue lines) for contacts made by the heavy (A) and light (B) chains were identified and visualized with LigPlot+ (R. A. Laskowski and M. B. Swindells, J Chem Inf Model 51:2778–2786, 2011, https://doi.org/10.1021/ci200227u). Residues contributing to hydrophobic interactions are shown as curved lines with dashes. Atoms in residues participating in hydrogen bonding are depicted as circles, with oxygen in red, carbon in black, and nitrogen in blue. Interacting residues that belong to complementarity-determining region (CDR) loops are colored as described in the legend to Fig. 3 in the main text. The antibody-antigen interface occludes approximately ∼1,200 Å^2^ of solvent-accessible surface area. Download FIG S3, TIF file, 2.2 MB. Copyright © 2021 Rissanen et al. 2021 Rissanen et al. https://creativecommons.org/licenses/by/4.0/ This content is distributed under the terms of the Creative Commons Attribution 4.0 International license.

10.1128/mBio.02531-20.4 FIG S4 Key interactions at the Fab nnHTN-Gn2–HTNV Gn complex interface. Paratope and epitope residues involved in hydrogen bonding (dashed black lines) and hydrophobic interactions (dashed blue lines) were identified and visualized with LigPlot+ (R. A. Laskowski and M. B. Swindells, J Chem Inf Model 51:2778–2786, 2011, https://doi.org/10.1021/ci200227u). Residues contributing to hydrophobic interactions are shown as curved lines with dashes. Atoms in residues participating in hydrogen bonding are depicted as circles, with oxygen in red, carbon in black, and nitrogen in blue. Interacting residues that belong to complementarity-determining region (CDR) loops are colored as described in the legend to Fig. 3 in the main text. The antibody-antigen interface occludes approximately 930 Å^2^ of solvent-accessible surface area. Download FIG S4, TIF file, 1.6 MB. Copyright © 2021 Rissanen et al. 2021 Rissanen et al. https://creativecommons.org/licenses/by/4.0/ This content is distributed under the terms of the Creative Commons Attribution 4.0 International license.

The epitope of the neutralizing Fab HTN-Gn1 occludes approximately 1,200 Å^2^ of solvent-accessible surface area and is reinforced by 18 hydrogen bonds and a salt bridge, identified by PDBePISA (53) and LigPlot+ (54). The majority of antibody complementarity-determining regions (CDRs), including CDRH1, CDRH3, CDRL2, and CDRL3, bind loop_β4-β5_. The epitope at loop_β4-β5_ contributes approximately 60% of the total buried surface, where the paratope partially wraps around the residues constituting the CDRs in the heavy chain. Although the epitope is predominantly protein specific, CDRH2 also forms minor contacts with the first and second *N*-acetylglucosamine (GlcNAc) moieties of the N-linked glycan chitobiose core extending from Asn134. The epitope of the non-neutralizing Fab nnHTN-Gn2 is slightly smaller, occluding approximately 930 Å^2^ of solvent-accessible surface area, and reinforced by 17 hydrogen bonds. Further detail for both antibody-antigen interfaces is presented in Fig. S3 and S4.

### HTN-Gn1-mediated targeting of the higher-order glycoprotein lattice.

**FIG 4:**
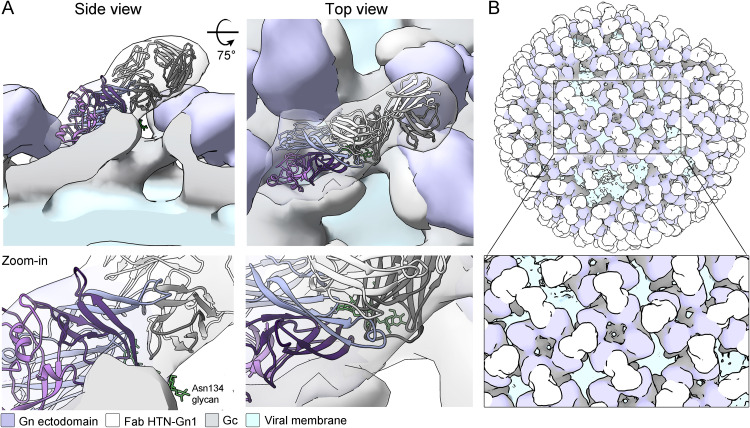
Cryo-ET of HTNV VLPs in complex with Fab HTN-Gn1 provides a model for mAb-mediated obstruction of the (Gn-Gc)_4_ lattice. (A) Side (left) and top (right) views of the HTNV VLP−Fab HTN-Gn1 reconstruction with the crystal structure of HTNV Gn−Fab HTN-Gn1 (cartoon representation and colored as described in the legend to Fig. 3) fit into the density as a single rigid body. The HTNV VLP is shown as a surface with density corresponding to Fab HTN-Gn1 colored white, the N-terminal ectodomain of HTNV Gn colored purple, the viral membrane colored light blue, and the expected ectodomain regions of the HTNV Gc colored gray. (B) Model of Fab HTN-Gn1 binding in the context of a HTNV VLP, prepared by mapping (Gn-Gc)_4_ spike complexes onto the refined coordinates of a single VLP in the data set. For each position, one of the two possible overlapping binding sites was chosen randomly. Colored as described for panel A.

To determine the mode by which Fab HTN-Gn1 interacts with the mature hantavirus spike, we performed cryo-ET on HTN virus-like particles (VLPs). HTN VLPs, produced via the expression of the HTNV GPC in mammalian tissue culture as previously described (33, 55), exhibit the classical hantaviral lattice comprised of interlinked tetrameric spikes (Fig. S5 and S6). While no major alterations to spike ultrastructure were observed following treatment with Fab HTN-Gn1 within the 1.9-nm resolution afforded by the method, additional density corresponding to the Fab protrudes from the surface of the membrane-distal tetrameric lobes. Indeed, fitting of our HTNV Gn−Fab HTN-Gn1 complex crystal structure into the Fab HTN-Gn1-treated VLP reconstruction resulted in an excellent fit (correlation coefficient, 0.9), which unambiguously reveals the orientation of the Gn in this region of the spike complex, and reveals that the HTN-Gn1 epitope is proximal to the Gn-Gc interface (Fig. 4A).

10.1128/mBio.02531-20.5 FIG S5 Additional density is observed on HTNV VLP surface after Fab HTN-Gn1 treatment. The tetragonal glycoprotein lattice organization is observed in HTNV VLP (12.3 Å) and in HTNV VLP treated with HTN-Gn1 (19 Å). Additional density at the tetrameric lobes is observed following Fab HTN-Gn1 treatment. The refinement of the Fab-bound reconstruction was focused on the rightmost Fab, resulting in complete density for this Fab fragment. Only partial density is visible for the other three Fab fragments, as these represent a superposition of symmetry-mismatched Fab fragments bound to one of two possible sites on the glycoprotein lattice. The top view of each viral surface reconstruction is shown, and densities corresponding to viral membrane and envelope glycoproteins are rendered light blue and white, respectively. Download FIG S5, TIF file, 2.1 MB. Copyright © 2021 Rissanen et al. 2021 Rissanen et al. https://creativecommons.org/licenses/by/4.0/ This content is distributed under the terms of the Creative Commons Attribution 4.0 International license.

10.1128/mBio.02531-20.6 FIG S6 Representative EM images of HTNV VLPs in the presence and absence of Fab HTN-Gn1. (A to D) Tomographic slices of HTNV VLP alone. (E to H) Tomographic slices of HTNV VLP in the presence of HTN-Gn1. Scale bars, 100 nm. Download FIG S6, TIF file, 2.7 MB. Copyright © 2021 Rissanen et al. 2021 Rissanen et al. https://creativecommons.org/licenses/by/4.0/ This content is distributed under the terms of the Creative Commons Attribution 4.0 International license.

**FIG 5:**
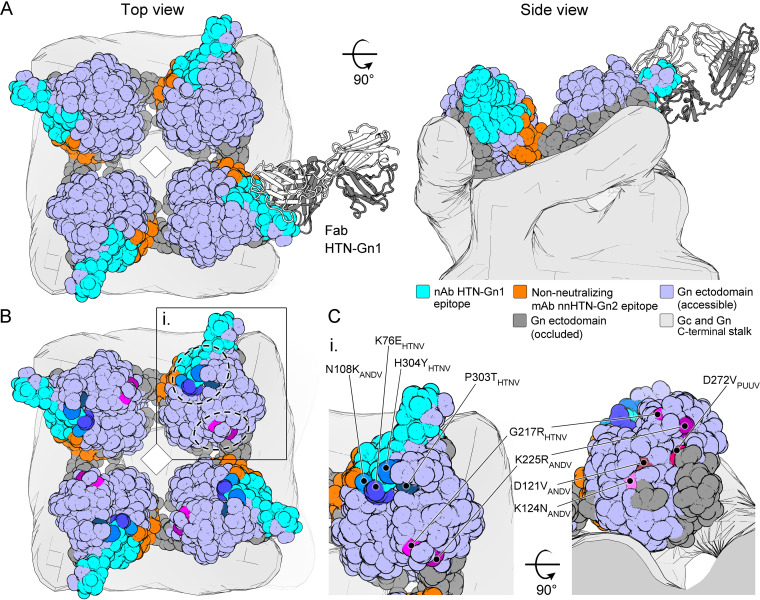
Epitope accessibility provides a rationale for neutralizing activity. (A) Mapping antibody-accessible surfaces onto the N-terminal region of HTNV Gn. The tetrameric assembly of HTNV Gn, formed upon fitting of the HTNV Gn−Fab HTN-Gn1 crystal structure into the HTNV-VLP reconstruction (Fig. 4), is shown in surface representation. Solvent-accessible surfaces of the HTNV Gn ectodomain are colored purple, occluded surfaces (with subunit contacts located within ≤10 Å) are colored dark gray, and mAb HTN-Gn1 and nnHTN-Gn2 epitopes are colored cyan and orange, respectively. Consistent with the non-neutralizing activity of mAb nn-HTN-Gn2, the nnHTN-Gn2 epitope is located at regions of the molecule expected to form intersubunit (i.e., Gn-Gn and Gn-Gc) interactions and is less immunogenically accessible than the neutralizing mAb HTN-Gn1 epitope in the context of the observed higher-order (Gn-Gc)_4_ lattice. (B) Mapping of neutralization evasion (NE) mutation sites that indicate key residues for neutralizing antibody activity, reported in the Gn ectodomain across hantaviral species, reveals that NE sites cluster at two regions on the Gn (highlighted in subunit [i.] by dashed circles). The epitope of mAb HTN-Gn1 colocalizes with one of these sites. (C) A close-up of a single Gn ectodomain subunit (i). The region proximal to mAb HTN-Gn1 epitope is critical to the activity of HTNV-neutralizing antibodies mAb 3D5 (NE mutations H304Y and K76E), 16E6 (P303T), and 16D2 (K76E) and ANDV-neutralizing mAb KL-AN-4E1 (N108K) (25, 27, 32). Details of the mapped NE sites are presented in Table S3.

This fitting allowed us to glean several insights into mAb recognition as well as the (Gn-Gc)_4_ assembly. First, this fitting rationalizes the approximate 50% occupancy of Fab HTN-Gn1 within the HTN VLP reconstruction, where Fab binding occludes the epitope of the adjacent Gn molecule in a neighboring spike (Fig. 4B). Second, the fitting is in good agreement with the previously proposed organization of hantaviral envelope (35), where the N-terminal ectodomain of the Gn contacts and likely stabilizes the prefusion conformation of Gc at the membrane-distal region of the (Gn-Gc)_4_ spike. Furthermore, consistent with its non-neutralizing functionality, this fitting reveals that the mAb nnHTN-Gn2 epitope is not natively accessible on the mature virus surface (Fig. 5).

10.1128/mBio.02531-20.9 TABLE S3 Known neutralization evasion mutation sites on the Gn component of the hantaviral Gn-Gc spike. The sites described below are illustrated in Fig. 5 in the main text (M. Kikuchi, K. Yoshimatsu, J. Arikawa, R. Yoshida, Y. C. Yoo, Y. Isegawa, K. Yamanishi, S. Tono-oka, and I. Azuma, Arch Virol 143:73–83, 1998, https://doi.org/10.1007/s007050050269; M. Wang, D. G. Pennock, K. W. Spik, and C. S. Schmaljohn, Virology 197:757–766, 1993, https://doi.org/10.1006/viro.1993.1652; J. Hörling and A. Lundkvist, Virus Res 48:89–100, 1997, https://doi.org/10.1016/s0168-1702(97)01436-6; J. Duehr, M. McMahon, B. Williamson, F. Amanat, A. Durbin, D. W. Hawman, D. Noack, S. Uhl, G. S. Tan, H. Feldmann, and F. Krammer, mBio 11:e00028-20, https://doi.org/10.1128/mBio.00028-20). Download Table S3, DOCX file, 0.02 MB. Copyright © 2021 Rissanen et al. 2021 Rissanen et al. https://creativecommons.org/licenses/by/4.0/ This content is distributed under the terms of the Creative Commons Attribution 4.0 International license.

Mutagenesis and peptide mapping studies have identified putative neutralizing epitopes on the hantaviral Gn that are likely targeted by the antibody-mediated immune response (25, 27–32). Mapping of all the neutralization evasion (NE) mutation sites reported in hantaviral Gn proteins revealed two regions that are key to the binding of neutralizing antibodies (Fig. 5). The mAb HTNV-Gn1 epitope overlaps with one of these sites, which is targeted by four known neutralizing antibodies: mAbs 3D5, 16E6, and 16D2, which neutralize HTNV (25, 32), and mAb KL-AN-4E1, which neutralizes ANDV (27) (Table S3). Furthermore, a peptide mimicking the PUUV loop_β4-β5_ was previously observed to react with serum samples from PUUV-infected patients (28), indicating that antibodies against this region are also produced upon zoonotic infection. As expected, none of the mapped NE sites overlap with the epitope of our non-neutralizing mAb nnHTN-Gn2.

## DISCUSSION

Given the widespread prevalence and considerable biomedical impact of hantaviruses upon human health, there is need for effective hantavirus vaccines. Several vaccine approaches based upon targeting hantaviral Gn and Gc are under development, including inactivated-virus vaccines (56, 57), virus-vectored recombinants (15, 58, 59), and DNA-based vaccines encoding the full M segment (21, 51). Of individuals in China receiving Hantavax, a formalin-inactivated vaccine prepared in mouse brains, 50% exhibited measurable nAb titers 1 year after vaccination (56, 57). A vesicular stomatitis virus (VSV)-based vaccine bearing the ANDV M segment generated a nAb response that protects against both ANDV and SNV infection in a hamster challenge model (60). Vaccines using other viral vectors, e.g., vaccinia virus, have faced challenges associated with pre-existing immunity to the vectors (59). More promisingly, phase 1 and 2a trials have been conducted with M segment DNA-based HTNV and PUUV vaccines in healthy volunteers, where nAbs were detected in up to 78% of volunteers when the vaccine was delivered intramuscularly by electroporation (61).

Here, we interrogated the molecular basis for targeting the hantaviral Gn, a central target of the neutralizing antibody response arising during infection (14, 15). Using a minimal subunit immunization approach, we show that the membrane-distal N-terminal ectodomain of recombinantly derived and purified, soluble hantaviral Gn (amino acid residues 18 to 371) can elicit nAbs in an animal model (Fig. 1 and 2). Although we envisage room for improvement and that future immunogens will elicit an increased nAb response, our HTNV Gn exhibited higher neutralization titers than those of a previous study utilizing a recombinant Gn immunogen, and this may be due to the different Gn coding region used (15). Similarly, adenovirus-vectored ANDV Gn (and Gc) alone did not elicit an immune response, whereas the full-length M segment did (62). Together, these observations are suggestive that the success of Gn and Gc subunit approaches may be dependent on protein integrity and presentation when in the absence of their cognate glycoprotein. mAb HTN-Gn1 modestly neutralized live virus *in vitro* with an IC_50_ of 15.47 μg/ml, which is within the range of IC_50_ values observed for anti-hantavirus mAbs obtained from hamsters (with IC_50_s ranging from 0.25 to 20.97 μg/ml for 19 hamster mAbs) (27) and humans (0.205 μg/ml and 6.60 μg/ml for two human mAbs) (23). Interestingly, selected mAbs within these libraries were shown to be sufficient to confer complete protection in a live virus animal challenge model, including a nAb (termed KL-HAP-6B12) that is more weakly neutralizing than HTN-Gn1 (27).

Our integrated cryo-ET and X-ray crystallography analyses of mAbs HTN-Gn1 and nnHTN-Gn2 identify regions of hantaviral Gn that are immunologically accessible in the context of the mature spike assembly displayed on the hantavirus surface (Fig. 4 and 5). Indeed, we find that neutralizing mAb HTN-Gn1 recognizes loop_β4-β5_ at an immune-accessible region that is known to react with the sera of PUUV-infected patients (28). Interestingly, loop_β4−β5_ appears to crystallize in variable configurations and exhibits conformational flexibility, as seen in the two structures reported here and in both previously reported hantavirus Gn crystal structures (34, 35). As the β4-β5 loop is proximal to the Gn-Gc interface, we speculate that the inherent plasticity of this region may reflect the requirement for a binding partner, such as the Gc, as observed in the crystal structure of the heterodimeric Gn-Gc complex reported by Serris et al. (36). We note that neutralizing mAb HTN-Gn1 binds to the epitope presented by recombinantly derived monomeric Gn, as well as Gn incorporated into the mature hantaviral surface, despite the likely differences in the conformation of the loop between these states. In contrast to the accessible mAb HTN-Gn1 epitope, non-neutralizing mAb nnHTN-Gn2 binds to a region of the Gn predicted to be sequestered within the higher-order (Gn-Gc)_4_ spike lattice (Fig. 5). Such information provides an important blueprint for the engineering of optimized immunogens capable of immunofocusing the antibody response to natively accessible surfaces bearing neutralizing epitopes and concealing off-target surfaces (e.g., through stable complexation with the cognate Gc or the addition of N-linked glycosylation) that are inaccessible in the context of the higher-order spike assembly. Interestingly, we note that the epitope targeted by mAb HTN-Gn1 is proximal to the binding site predicted for a mAb (mAb KL-AN-4E1) identified by Duehr et al. (27). Furthermore, several other epitopes from this study also map to this membrane-distal surface of the hantavirus envelope, indicating that this region of the hantaviral Gn may constitute a commonly targeted epitope by the nAb response (Fig. 5; see Table S3 in the supplemental material). Given that HTN-Gn1 is capable of occluding much of the protein surface (Fig. 4B), it seems possible that our nAb interferes with host cell recognition, similar to what has been observed for Rift Valley fever virus (RVFV) Gn-specific nAbs (63). Further, although we cannot preclude the possibility that mAb HTN-Gn1 induces virus aggregation through cross-linking of virions via available Fabs, the high density of HTN-Gn1 epitopes on the virus surface (Fig. 4 and 5) supports a model whereby both Fab regions of mAb HTN-Gn1 are able to recognize the same virion simultaneously. Future mechanistic studies will no doubt shed light on the common or differing mechanism(s) of neutralization that may result from targeting this region of the molecule.

Although the use of the soluble N-terminal region of the hantaviral Gn ectodomain as an immunogen has not been previously reported, there is precedent for adopting such an approach in related bunyaviral families. Indeed, immunization with an N-terminal region of the Gn ectodomain from RVFV (family *Phenuiviridae*) has been shown to elicit neutralizing antibodies in rabbits (52). Similarly, immunization with an N-terminal region of the Gc ectodomain from Schmallenberg virus (family *Peribunyavirus*), which appears to provide a similar structural and shielding role to the hantaviral and phleboviral Gn, results in a neutralizing antibody response that protects against infection in mice (64). Although the structure and display of these regions of Gn and Gc within higher order Gn-Gc assemblies are highly distinctive, they are united in their distal placement from the virion membrane (35, 36, 41, 64, 65). Future studies should focus on comparing the differential antigenicities of individual regions of bunyaviral Gn and Gc ectodomains in the context of their varied levels of immune accessibility on the mature virion surface. Such comparisons will likely inform immunofocused approaches that enhance the nAb response generated upon immunization.

Recent major outbreaks of severe acute respiratory syndrome coronavirus 2 (SARS-CoV-2), Zika virus, and Ebola virus demonstrate the acute threat that emerging viruses pose to human health and economy (66–68). As with these prominent pathogens, the emergence and severe disease caused by HTNV and other hantaviruses necessitate an improved understanding of virus pathobiology and the development of new strategies to prevent and respond to infection. By dissecting the tetrameric hantaviral (Gn-Gc)_4_ spike and demonstrating that the recombinant HTNV Gn subunit elicits an antibody response that neutralizes HTNV, our work both refines the antigenic topography of the hantaviral surface and validates the N-terminal region of the hantaviral Gn glycoprotein as a potential target for rational vaccine development efforts.

## MATERIALS AND METHODS

### Production of recombinant HTNV Gn.

The N-terminal ectodomain of HTNV Gn was expressed and purified as previously described (34). Briefly, codon-optimized synthetic Gn cDNA (GeneArt, Life Technologies) coding for Gn residues 18 to 371 (GenBank accession number AIL25321.1) was cloned into the pHLsec mammalian expression vector (69) and transiently expressed in human embryonic kidney (HEK) 293T cells (ATCC CRL-3216), in the presence of the class 1 α-mannosidase inhibitor kifunensine (69, 70) at 5 μM concentration. Diafiltrated cell supernatant was purified by immobilized metal affinity chromatography (5 ml Fast Flow [FF] crude column and ÄKTA fast protein liquid chromatography [FPLC] system; GE Healthcare), followed by size exclusion chromatography using a Superdex 200 10/300 increase column (GE Healthcare), in 10 mM Tris (pH 8.0), 150 mM NaCl buffer.

### Animal immunization.

The rabbit immunization study was approved and carried out in accordance with protocols provided to the Institutional Animal Care and Use Committee (IACUC) at Scripps Research (La Jolla, CA) under approval number 14-0002-2. The rabbits were kept, immunized, and bled at Scripps Research in compliance with the Animal Welfare Act and other federal statutes and regulations relating to animals and in adherence to the *Guide for the Care and Use of Laboratory Animals* (71). Four female, 12-week-old New Zealand White rabbits were used in immunization studies.

Rabbits were primed (subcutaneously [s.c.]) with purified HTNV Gn ectodomain (100 μg) adjuvanted with the Sigma adjuvant system or alum (Sigma-Aldrich) at a ratio of 1:5 of adjuvant to immunogen in sterile phosphate-buffered saline (PBS) (1-ml total volume). Following immunization, a further two boosts were conducted at 4-week intervals. Sera were prepared from blood collected prior to immunization and 7 days following each immunization/boost. PBMCs were isolated using a Lymphoprep (Stemcell Technology) density gradient and cryopreserved in fetal bovine serum (FBS) plus 10% dimethyl sulfoxide (DMSO).

### B cell sorting.

Fluorescence-activated cell sorting of cryopreserved PBMCs was performed on a BD Aria II. PBMCs were stained with anti-rabbit-CD3–phycoerythrin (PE) (Santa Cruz Biotechnology), anti-rabbit-IgM–fluorescein isothiocyanate (FITC) (Southern Biotech), anti-rabbit-IgG–Alexa Fluor 647 (Southern Biotech), and biotinylated HTNV-Gn incubated with streptavidin-peridinin chlorophyll protein (PerCP)-Cy5.5 (BD). CD3^−^ IgM^−^ IgG^+^ HTNV Gn^+^ cells were sorted into individual wells containing RNase OUT (Invitrogen), first-strand SuperScript III buffer, dithiothreitol (DTT), and H_2_O (Invitrogen), and RNA was converted into cDNA (SuperScript III reverse transcriptase; Invitrogen) using random hexamers in accordance with the manufacturer’s protocol.

### Full-length antibody cloning and expression.

The rabbit Ab variable regions of heavy and kappa chains were PCR amplified using previously described primers and PCR conditions (52). PCR products were purified and cloned into an expression plasmid adapted from the pFUSE-rIgG-Fc and pFUSE2-CLIg-rK1 vectors (InvivoGen) using the Gibson Assembly master mix (NEB) under ampicillin selection by following the manufacturer’s protocol. Ab variable regions were sequenced by Sanger sequencing.

Ab heavy and light plasmids were cotransfected at a 1:1 ratio into HEK 293F cells (Thermo Fisher) using PEI Max 40K (linear polyethylenimine hydrochloride; Polysciences, Inc.). Ab supernatants were harvested 5 days following transfection and purified using protein G affinity chromatography by following the manufacturer’s protocol (GE Healthcare).

### Fab production and purification.

Fab fragments from mAbs HTN-Gn1 and nnHTN-Gn2 were expressed from codon-optimized synthetic cDNA (GeneArt, Life Technologies) templates. Synthetic DNA encoding light and heavy chains of each mAb were individually cloned into the pHLsec (69) mammalian expression vector. Light and heavy chain pairs were then cotransfected into HEK 293T cells (ATCC CRL-3216) for transient expression as previously described, using 0.5 mg heavy-chain and 0.5 mg light-chain plasmid DNA per liter of cell culture in transfection and polyethylenimine as the transfection reagent (69). Expression conditions also included the class 1 α-mannosidase inhibitor kifunensine at 5 μM concentration (69, 70). At 96 h post-transfection, Fab-containing supernatants were collected, clarified by centrifugation, and diafiltered using the ÄKTA Flux tangential flow filtration system. Diafiltered cell supernatants were purified by immobilized metal-affinity chromatography (5 ml FF crude column and ÄKTA FPLC system; GE Healthcare) at room temperature, using 250 mM imidazole for elution, followed by size exclusion chromatography using a Superdex 200 10/300 increase column (GE Healthcare), in 10 mM Tris (pH 8.0), 150 mM NaCl buffer.

### Ab binding ELISA.

ELISAs were carried out as previously described (72). High-binding ELISA 96 half-well microplates (Corning) were coated with purified HTNV Gn (25 μl, 3 μg/ml in PBS) overnight at 4°C. Plates were washed five times with PBS containing 0.05% Tween 20 (PBS-T) and blocked with blocking buffer (5% nonfat milk in PBS-T) for 1 h at room temperature (RT). The blocking buffer was removed, and serially diluted Ab (starting at 20 μg/ml, 1:5 dilution in blocking buffer) or serum (starting at 1:50, 1:5 dilution in blocking buffer) was added for 2 h at RT. Plates were washed five times with PBS-T. Secondary Ab [goat anti-rabbit IgG F(ab′)_2_, AP conjugate; Invitrogen; 1:1,000] was added for 1 h, and the plates were washed as described above. The *p*-nitrophenyl phosphate substrate (Sigma) was added to detect binding, and the optical densities (ODs) were measured at 405 nm.

### Viruses, cells, and medium.

HTNV strain 76-118 was kindly provided by J. Hooper and propagated in African green monkey kidney cells (Vero E6) that were kindly provided by J. L. Whitton. Vero E6 cells were maintained in complete Dulbecco’s modified Eagle medium (cDMEM) (11965-092) containing 10% fetal bovine serum (FBS) (16140-071), 1% HEPES buffer solution (15630-130), and 1% penicillin-streptomycin (15140-122) (all from Thermo Fisher Scientific, Carlsbad, CA). Cells were grown in a humidified incubator at 37°C with 5% CO_2_.

### Focus reduction neutralization test.

To assess the neutralization capacity of the rabbit sera or monoclonal IgG antibodies HTN-Gn1 and nnHTN-Gn2 against HTNV strain 76-118, we conducted a focus reduction neutralization test (FRNT) in the UVM BSL-3 facility under an approved Institutional Biosafety Protocol. Each antibody was diluted serially in 50 μl of cDMEM, mixed with an equal volume of cDMEM containing 100 focus-forming units (FFU) of HTNV, and then incubated for 60 min at 37°C. The medium from confluent Vero E6 cell monolayers in 48-well tissue culture plates was removed, and 100 μl of the antibody-virus mixture was inoculated onto the cells and incubated at 37°C in a 5% CO_2_ incubator for 60 min, after which the wells were overlaid with 1.2% methylcellulose in cDMEM and incubated at 37°C in a 5% CO_2_ incubator for 10 days. Infected cells were fixed in 25% formaldehyde in 3× phosphate-buffered saline (PBS). Cells were permeabilized with 0.1% 100× Triton in 1× PBS for 15 min and then incubated with the primary rabbit anti-ANDV N polyclonal antibody (NR-12152; BEI Resources) (1:20,000), followed by a peroxidase-labeled goat anti-rabbit antibody (5220-0336; SeraCare) (1:2,000) and then the peroxidase substrate (5510-0030; SeraCare). Images of the wells were captured using an Alpha Innotech imager, and viral foci were quantified manually. For the experiments illustrated in Fig. 2B and D, sera or mAbs were screened in two biological replicates, where each biological replicate featured two technical replicates.

### Fab-HTNV Gn complex preparation, crystallization, and structure determination.

Purified Fab HTN-Gn1 and Fab nnHTNV-Gn2 samples were individually mixed with purified HTNV Gn at a stoichiometry of 1.2:1. To aid crystallogenesis, high-mannose-type N-linked glycans resulting from expression in the presence of kifunensine were trimmed by partial enzymatic deglycosylation with endoglycosidase F1 (73). The endoglycosidase was added to Fab-Gn mixtures at a 1:100 (wt/wt) ratio, and samples were incubated for 18 h at room temperature. Deglycosylated Fab-Gn mixtures were purified by size exclusion chromatography using a Superdex 200 10/300 increase column (GE Healthcare), in 10 mM Tris (pH 8.0), 150 mM NaCl buffer. Elution peaks corresponding to 1:1 Fab-Gn complexes were collected and concentrated for crystallization.

Samples of Gn−Fab HTN-Gn1 (7.2 mg/ml) and Gn−Fab nnHTN-Gn2 (5.3 mg/ml) were crystallized at room temperature with the sitting-drop vapor diffusion method using 100 nl protein in 10 mM Tris (pH 8.0), 150 mM NaCl buffer plus 100 nl precipitant. Diffracting crystals of Gn−Fab HTN-Gn1 were obtained from a Morpheus screen (74) condition comprising 0.1 M HEPES/MOPS (morpholinepropanesulfonic acid) (pH 7.5), 0.12 M monosaccharides (glucose, mannose, galactose, fucose, xylose, *N*-acetylglucosamine [NAG]), and 30% (wt/vol) ppt2 (ethylene glycol and polyethylene glycol [PEG] 8000), while diffracting crystals of Gn−Fab nnHTN-Gn2 grew in a polyglutamic acid (PGA) screen (Molecular Dimensions) condition comprising 0.1 M sodium cacodylate (pH 6.5), 5% (wt/vol) polyglutamic acid low molecular weight (PGA-LM), and 20% (wt/vol) PEG 3350.

Crystals were cryoprotected by being briefly soaked in reservoir solution supplemented with 25% (vol/vol) glycerol before flash-cooling in liquid nitrogen. X-ray diffraction data were recorded on a Dectris PILATUS3 6M detector at beamline i03 of Diamond Light Source, United Kingdom, at wavelengths (λ) of 0.9762 Å and 0.9686 Å for Gn−Fab HTN-Gn1 and Gn−Fab nnHTN-Gn2, respectively. Data were indexed, integrated, and scaled with XIA2 using the DIALS pipeline (75–77). Both structures were solved by molecular replacement with the program Phaser-MR within the PHENIX suite (78). Search models used in molecular replacement comprised Fab homology models generated using the SWISS-MODEL server (79) for Fabs HTN-Gn1 and nnHTN-Gn2 and the previously reported HTNV Gn structure (PDB ID 5OPG). Iterative structure refinement was performed on both models using REFMAC (80–82) and PHENIX (78). Coot (83) was used for manual rebuilding, and MolProbity (84) was used to validate models. Processing statistics are presented in Table S2 in the supplemental material. Molecular graphics images were generated using PyMOL (The PyMOL Molecular Graphics System, version 1.7.0.3; Schrödinger, LLC) and UCSF Chimera (85).

### Preparation of HTNV VLPs.

HTNV virus-like particles (VLPs) were produced by transient expression of the complete HTNV M segment (GenBank accession number AIL25321.1), cloned into the pCAGGS vector, in HEK 293T cells. Six five-layer 875-cm^2^ flasks (Falcon) were used to produce 750 ml of VLP-containing medium that was clarified at 3,000 × *g* for 20 min to remove cell debris and filtered through a 0.45-μm filter. The virus-containing medium was concentrated to approximately 30 ml using a pump-powered filter (100-kDa cutoff) (Vivaflow; Sartorius) and then dialyzed into an excess of buffer (10 mM Tris [pH 8.0], 150 mM NaCl) through a 1-MDa-cutoff dialysis membrane (biotech CE tubing; Spectrum Chemical) for a few days. The medium was further concentrated to ∼3 ml with a 100-kDa-cutoff centrifugal concentrator filter (Amicon ultra; Merck Millipore) and layered onto a 20-to-50% (wt/vol) sucrose density gradient in PBS buffer. The gradient was prepared using a Gradient Master (BioComp Instruments, Canada) in an SW32 Beckman tube, and the VLPs were banded by ultracentrifugation at 4°C for 4 h at 25,000 rpm. The diffuse band (volume of ∼3 to 4 ml) was collected manually, diluted to 20 ml of PBS, and pelleted through a cushion of 10% sucrose in PBS to further clean and concentrate the sample (SW32 Beckman centrifuge tube at 25,000 rpm at 4°C for 2 h). Finally, the pellet was resuspended in 60 μl of PBS and stored at 4°C.

### Cryo-EM grid preparation, data acquisition, and data processing.

A 3-μl aliquot of VLP sample supplemented by 3 μl of 6-nm gold fiducial markers (Aurion) was applied to a holey carbon grid (2-μm hole diameter, C-flat; Protochips) that had been glow discharged in a plasma cleaner (Harrick) for 15 s. The grids were blotted for 3 to 4 s at 4°C and plunged into an ethane/propane mixture using a vitrification apparatus (Vitrobot; Thermo Fisher Scientific). For Fab HTN-Gn1-treated VLP electron microscopy (EM) sample preparation, a suspension of purified VLPs was incubated with 1.1 μM Fab HTN-Gn1 for 1 h at room temperature prior to grid preparation.

Data were collected using a Titan Krios transmission electron microscope (Thermo Fisher Scientific) operated at 300 kV and at liquid nitrogen temperature. Tomo4 software was used to acquire tomographic tilt data on a direct electron detector (K2 Summit; Gatan) mounted behind an energy filter (0 to 20 eV) (QIF Quantum LS; Gatan) (Table S4).

10.1128/mBio.02531-20.10 TABLE S4 Cryo-EM tomography data collection and subtomogram averaging statistics. Download Table S4, DOCX file, 0.02 MB. Copyright © 2021 Rissanen et al. 2021 Rissanen et al. https://creativecommons.org/licenses/by/4.0/ This content is distributed under the terms of the Creative Commons Attribution 4.0 International license.

Movie frames were aligned and averaged using MotionCor2 to correct for beam-induced motion (86). Contrast transfer function (CTF) parameters were estimated using CTFFIND4 (87), and a dose-weighting filter was applied to the images according to their accumulated electron dose as described previously (88). These preprocessing steps were carried out using a custom script named tomo_preprocess (available upon request). Tilt images were then aligned using gold fiducial markers, corrected for the effects of CTF by phase flipping, and used to reconstruct three-dimensional (3D) tomograms in IMOD (89). The amplitudes of the subvolumes cropped from the tomograms were weighted to correct for the low-pass filtering function resulting from dose-weighting of the original images using a custom script (available on request).

Subvolume averaging for apo-HTNV and HTN-Gn1-Fab bound data sets was performed in Dynamo (90) by following procedures established earlier (35, 91). Refinements were carried out using a map of the Tula virus (TULV) GP spike (EMD-4867) as an initial template. The template was low-pass filtered to a 50-Å frequency to avoid model bias. Overlapping particles were removed based on a distance filter (106 Å) and cross-correlation threshold after each iteration. A custom script (PatchFinder; available at https://github.com/OPIC-Oxford/PatchFinder) was used to restrict the data set to include only spikes that were part of a lattice based on a given set of tolerances (49-Å deviation in the position, 25 degrees in their orientation). Spikes were defined as being part of a lattice if they had at least three interacting neighbors. These steps were performed in an identical fashion for both the apo-HTNV and HTNV-Gn1-Fab data sets.

In the case of the apo-HTNV data set, refined coordinates were converted from data binned by a factor of 4 to data binned by a factor of 2 and then subjected to 3D classification in RELION (92) before further refinement in Dynamo. The final apo-HTNV reconstruction, comprised of 3,209 subvolumes, was filtered to 12.3 Å as determined by Fourier shell correlation (FSC) (0.143 threshold).

To resolve the variable occupancy of the bound Fab in the HTNV-Gn1-Fab data set, the coordinates centered at each spike were shifted to the interspike region. Classification was then performed in RELION with a tight mask around the density corresponding to the Fab without imposing symmetry or allowing image shifts. This enabled the orientation of the Fab in each class to be determined and then rotated to match the other classes before further classification. The HTN-Gn1-Fab reconstruction was calculated from 2,380 subvolumes by using particles binned by a factor of 4 without further refinement. The threshold value for the rendered isosurface was determined for both reconstructions according to the molecular weight of the viral ectodomain proteins and Fab fragments, assuming an average protein density of 0.81 Da/Å³. Subvolume averaging statistics are presented in Table S4.

### Fitting of the Gn−Fab HTN-Gn1 crystal structure into the cryo-EM reconstruction.

Initial placement of the Gn−Fab HTN-Gn1 crystal structure into the reconstruction of the Fab-decorated HTNV VLP glycoprotein envelope was obtained using the “dock in map” function within the PHENIX cryo-EM suite (78, 93). Protein chains comprising a single Gn−Fab HTN-Gn1 complex were fitted into the EM reconstruction by using a map simulated for the protein at a resolution of 19 Å, to match the resolution of the spike reconstruction. The minimum accepted correlation coefficient of the placement was set at 0.6, and rigid body refinement of the placement was performed with the structure as a single unit. The top solution localized the model in the EM reconstruction so that the Fab occupies the novel density observed on the VLP surface following Fab treatment. This fit was further optimized by the UCSF Chimera “fit in map” function (85) and yielded a correlation coefficient score of 0.9 (Table S4), indicating an excellent agreement between a map simulated for the protein at 19 Å with the cryo-EM reconstruction.

### Data availability.

Atomic coordinates and structure factors of Fab HTN-Gn1 and Fab nnHTN-Gn2 in complex with HTNV Gn have been deposited in the PDB (accession codes 7NKS and 7O9S, respectively). EM structures of the HTNV VLP surface alone (accession code EMD-12543) and in complex with Fab HTN-Gn1 (EMD-12544) have been deposited in the EMDB at the EBI. Coordinates of HTNV Gn−Fab HTN-Gn1 fitted into EMD-12544 have been deposited in the PDB (accession code 7NRH).
