# Supplementary material for: Structural Basis for a Neutralizing Antibody Response Elicited by a Recombinant Hantaan Virus Gn Immunogen
Source: mBio. 2021 Jul 6;12(4):e02531-20. doi: 10.1128/mBio.02531-20 (PMC8406324; doi:10.1128/mBio.02531-20)
Supplement: FIG S2 [file mbio.02531-20-sf002.pdf]

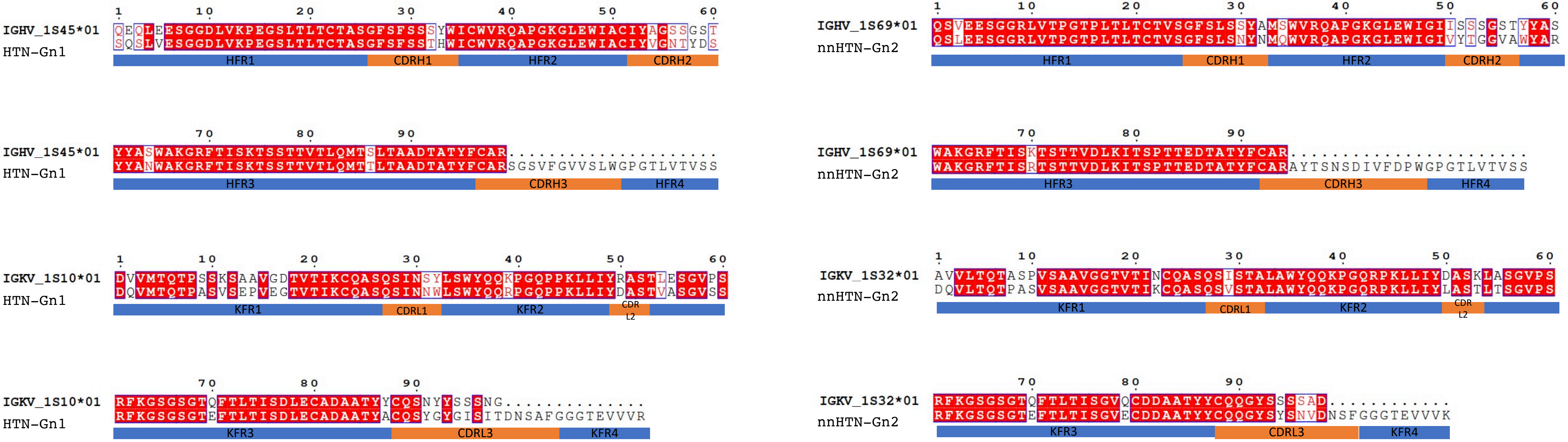
1 10 20 30 40 50 60
IGHV_1S45*01 QEQLEESGGDLVKPEGSLTLTCTASGFSFSSSYWICWVRQAPGKGLEWIACIYAGSSGST
HTN-Gn1 SQSLVESGGDLVKPEGSLTLTCTASGFSFSSTHWICWVRQAPGKGLEWIACIYVGNTYDS
HFR1 CDRH1 HFR2 CDRH2
70 80 90
IGHV_1S45*01 YYASWAKGRFTISKTSSTTVTLQMTSLTAADTATYFCAR..................
HTN-Gn1 YYANWAKGRFTISKTSSTTVTLQMTTLTAADTATYFCARSGSVFGVVSLWGPGTLVTVSS
HFR3 CDRH3 HFR4
1 10 20 30 40 50 60
IGKV_1S10*01 DVVMTQTPSSKSAAVGDTVTIKCQASQSINSYLSWYQQKPGQPPKLLIYRASTLESGVPS
HTN-Gn1 DQVMTQTPASVSEPVEGTVTIKCQASQSINNWLSWYQQRPGQPPKLLIYDASTVASGVSS
KFR1 CDRL1 KFR2 CDR L2
70 80 90
IGKV_1S10*01 RFKGSGSGTQFTLTISDLECADAATYYCQSNYYSSSNG..............
HTN-Gn1 RFKGSGSGTEFTLTISDLECADAATYACQSYGYGISITDNSAFGGGTEVVVR
KFR3 CDRL3 KFR4
1 10 20 30 40 50 60
IGHV_1S69*01 QSVEESGGRLVTPGTPLTLTCTVSGFSLSSYAMSWVRQAPGKGLEWIGIISSSGSTYYAS
nnHTN-Gn2 QSLEESGGRLVTPGTPLTLTCTVSGFSLSNYNMQWVRQAPGKGLEWIGIVYTGGVAWYAR
HFR1 CDRH1 HFR2 CDRH2
70 80 90
IGHV_1S69*01 WAKGRFTISKTSTTVDLKITSPTTEDTATYFCAR.....................
nnHTN-Gn2 WAKGRFTISRTSTTVDLKITSPTTEDTATYFCARAYTSNSDIVFDPWGPGTLVTVSS
HFR3 CDRH3 HFR4
1 10 20 30 40 50 60
IGKV_1S32*01 AVVLTQTASPVSAAVGGTVTINCQASQSISTALAWYQQKPGQRPKLLIYDASKLASGVPS
nnHTN-Gn2 DQVLTQTPASVSAAVGGTVTIKCQASQSVSTALAWYQQKPGQRPKLLIYLASTLTSGVPS
KFR1 CDRL1 KFR2 CDR L2
70 80 90
IGKV_1S32*01 RFKGSGSGTQFTLTISGVQCDDAATYYCQQGYSSSSAD............
nnHTN-Gn2 RFKGSGSGTEFTLTISGVECDDAATYYCQQGYSYSNVDNSFGGGTEVVVK
KFR3 CDRL3 KFR4
