# Supplementary figures and images for: Structural Basis for a Neutralizing Antibody Response Elicited by a Recombinant Hantaan Virus Gn Immunogen
Source: mBio. 2021 Jul 6;12(4):e02531-20. doi: 10.1128/mBio.02531-20 (PMC8406324; doi:10.1128/mBio.02531-20)

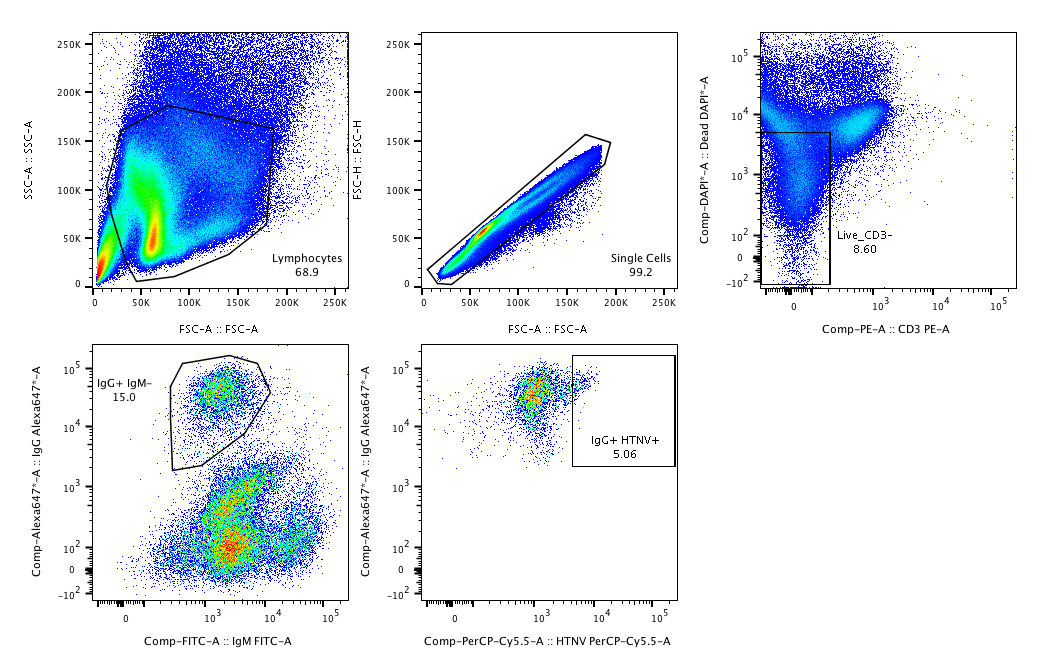

Supplement: FIG S1 [file mbio.02531-20-sf001.tif]

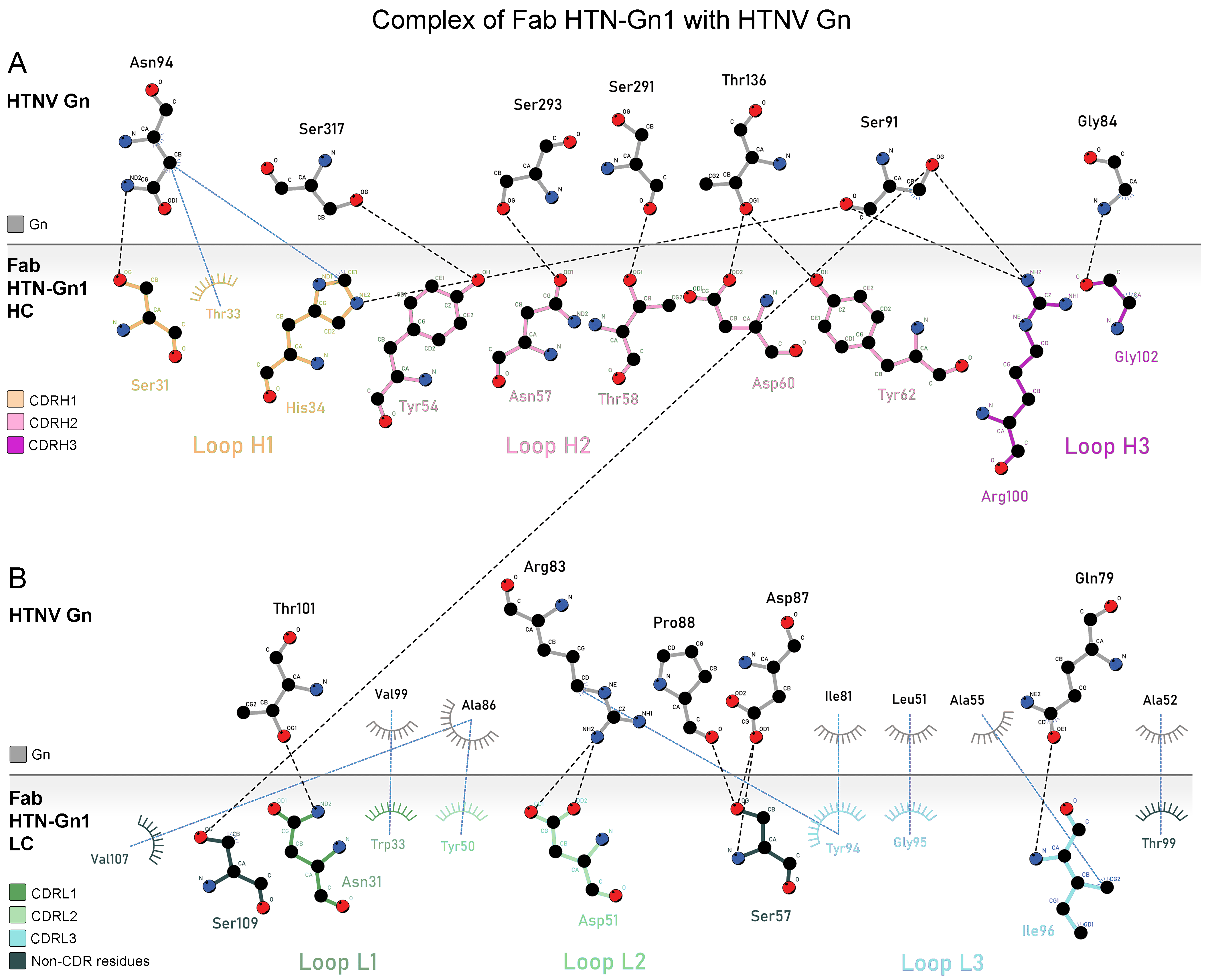

Supplement: FIG S3 [file mbio.02531-20-sf003.tif]

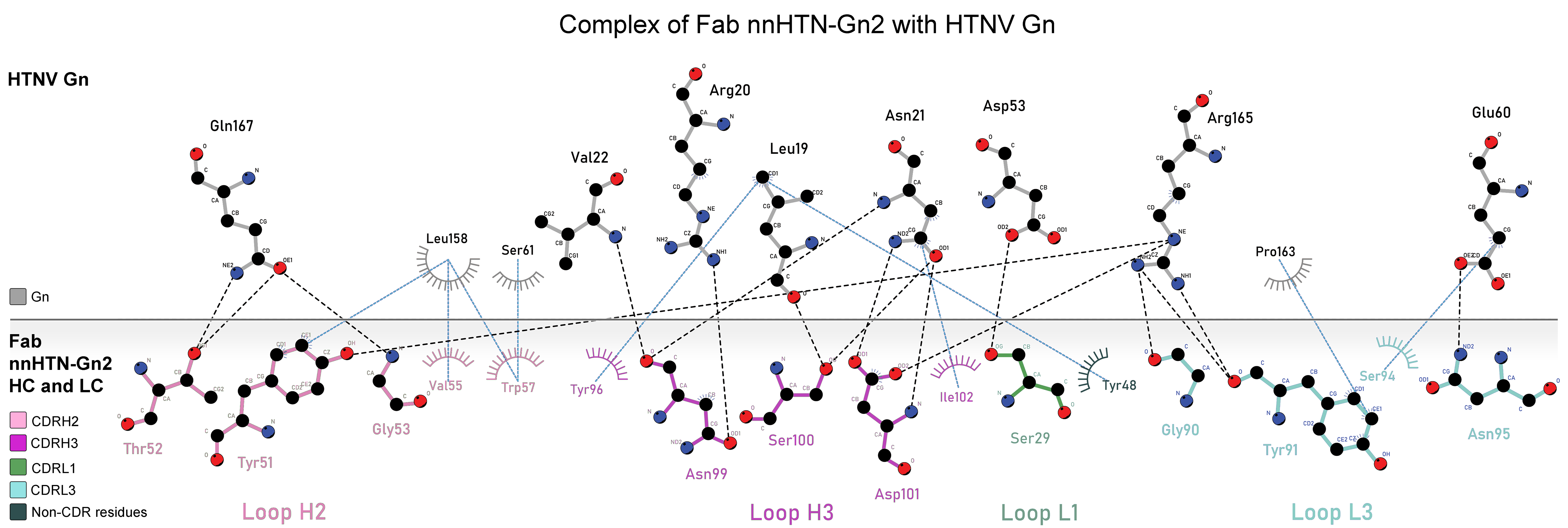

Supplement: FIG S4 [file mbio.02531-20-sf004.tif]

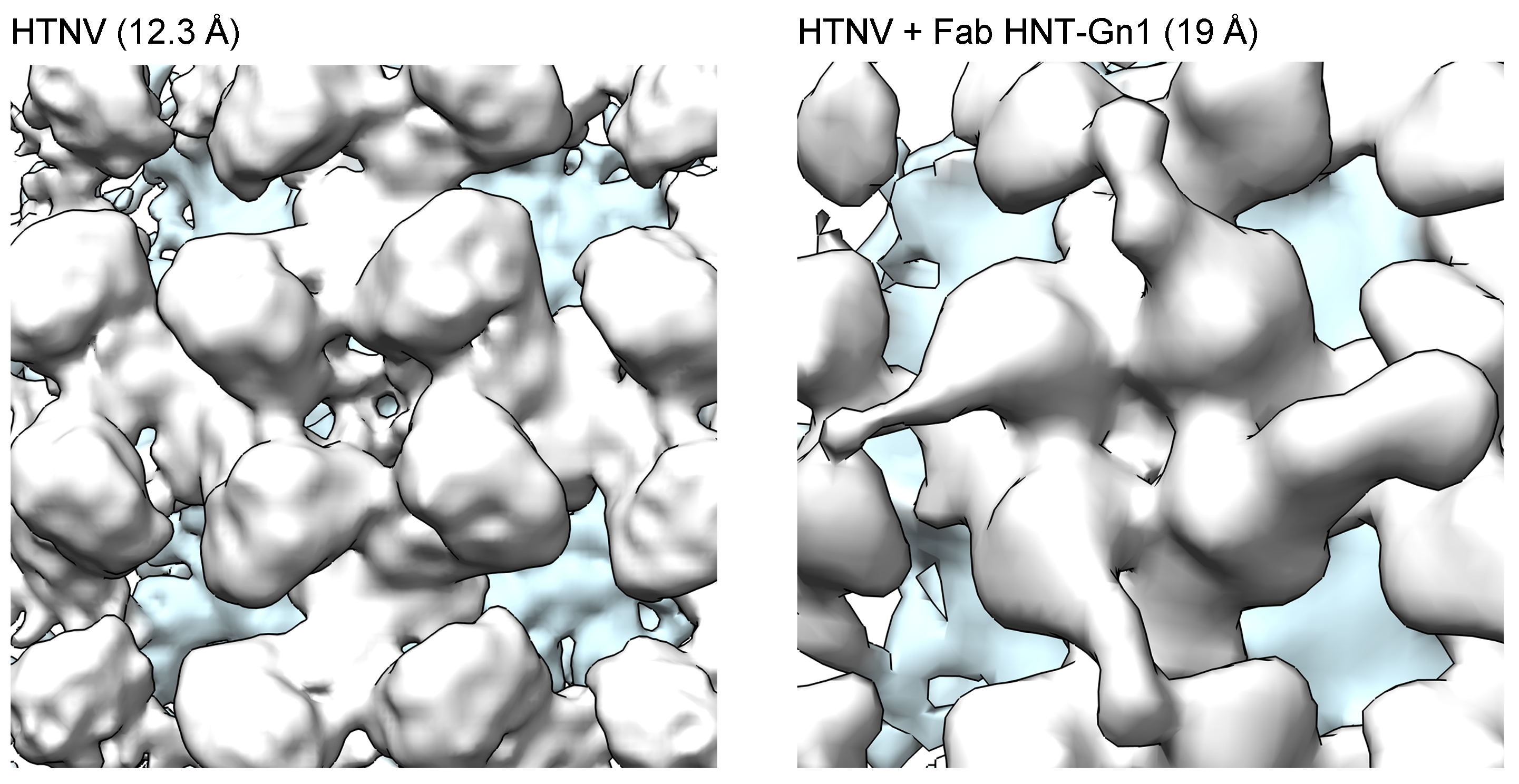

Supplement: FIG S5 [file mbio.02531-20-sf005.tif]

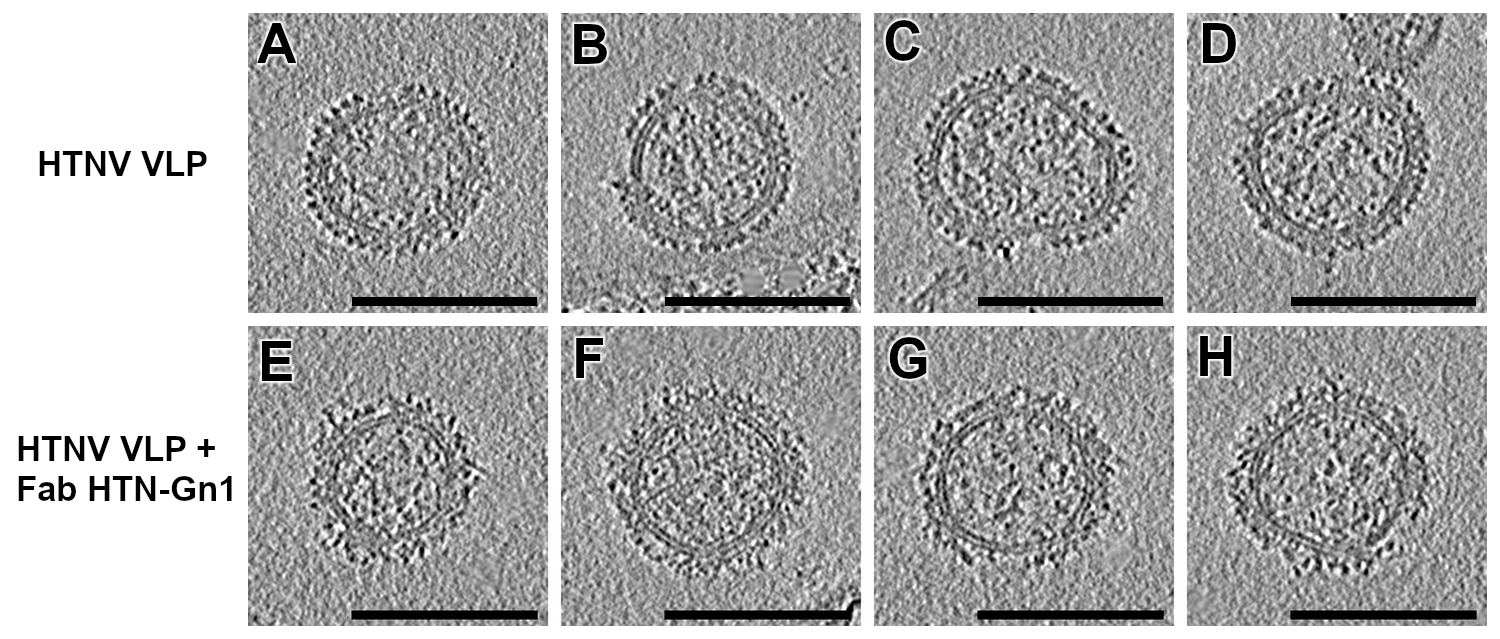

Supplement: FIG S6 [file mbio.02531-20-sf006.tif]
