# Supplementary material for: Structural Basis for a Neutralizing Antibody Response Elicited by a Recombinant Hantaan Virus Gn Immunogen
Source: mBio. 2021 Jul 6;12(4):e02531-20. doi: 10.1128/mBio.02531-20 (PMC8406324; doi:10.1128/mBio.02531-20)
Supplement: TABLE S1 [file mbio.02531-20-st001.docx]

| **Heavy** | **V region** | **Identity**  **(% nt)** | **J region** | **Identity (% nt)** | **D region** | **CDR1** | **CDR2** | **CDR3** |
| --- | --- | --- | --- | --- | --- | --- | --- | --- |
| HTN-Gn1 | Orycun IGHV1S45*01 F | 93.1 | Orycun IGHJ4*01 F | 70.8 | Orycun IGHD6-1*01 F | GFSFSSTHW | IYVGNTYDS | CARSGSVFGVVSLW |
| nnHTN-Gn2 | Orycun IGHV1S69*01 F | 90.6 | Orycun IGHJ2*01 F | 86.0 | Orycun IGHD1-1*01 F | GFSLSNYN | VYTGGVA | CARAYTSNSDIVFDPW |
|  |  |  |  |  |  |  |  |  |
| **Kappa** | **V region** | **Identity**  **(% nt)** | **J region** | **Identity (% nt)** | **D region** | **CDR1** | **CDR2** | **CDR3** |
| HTN-Gn1 | Orycun IGKV1S10*01 F | 88.9 | Orycun IGKJ1-2*02 F | 85.0 | N/A | QSINNW | DAS | CQSYGYGISITDNSAF |
| nnHTN-Gn2 | Orycun IGKV1S32*01 [F] | 93.2 | Orycun IGKJ1-2*01 F | 97.2 | N/A | QSVSTA | LAS | CQQGYSYSNVDNSF |
