# Supplementary material for: Structural Basis for a Neutralizing Antibody Response Elicited by a Recombinant Hantaan Virus Gn Immunogen
Source: mBio. 2021 Jul 6;12(4):e02531-20. doi: 10.1128/mBio.02531-20 (PMC8406324; doi:10.1128/mBio.02531-20)
Supplement: TABLE S2 [file mbio.02531-20-st002.docx]

|  | Gn−Fab HTN-Gn1 | Gn−Fab nnHTN-Gn2 |
| --- | --- | --- |
| **Data collection** |  |  |
| Space group | *P* 21 21 2 | *P* 21 21 21 |
| Cell dimensions |  |  |
| *a*, *b*, *c* (Å) | 228.0, 158.6, 70.9 | 76.1, 76.9, 174.4 |
| * α*, *β*, *γ * () | 90.0, 90.0, 90.0 | 90.0, 90.0, 90.0 |
| Resolution (Å) | 62.3-3.50 (3.56-3.50)* | 76.1-2.7 (2.75-2.7) |
| *R*_merge_ | 0.15 (0.67) | 0.08 (0.66) |
| I/σ I | 12.1 (1.9) | 19.9 (3.4) |
| CC_1/2_ | 0.99 (0.94) | 0.99 (0.96) |
| Completeness (%) | 100 (99.7) | 99.4 (98.9) |
| Multiplicity | 12.9 (11.5) | 13.1 (13.3) |
| **Refinement** |  |  |
| Resolution (Å) | 57.0-3.5 (3.6-3.5) | 51.7-2.7 (2.8-2.7) |
| No. reflections | 33,221 | 28,698 |
| *R*_work_ / *R*_free_ | 0.224 / 0.274 | 0.212 / 0.267 |
| No. atoms |  |  |
| Protein | 11,662 | 5,808 |
| Ligand/ion | 198 | 46 |
| Water | 0 | 0 |
| *B*-factors |  |  |
| Protein | 124.6 | 82.8 |
| Ligand/ion | 150.3 | 100.6 |
| Ramachandran plot (%) |  |  |
| Favored region | 96.27 | 97.36 |
| Allowed region | 3.73 | 2.64 |
| Outliers | 0 | 0 |
| R.m.s deviations |  |  |
| Bond lengths (Å) | 0.002 | 0.003 |
| Bond angles () | 0.556 | 0.692 |

* Values for the highest resolution shell are shown in parentheses
