# Supplementary material for: Structural Basis for a Neutralizing Antibody Response Elicited by a Recombinant Hantaan Virus Gn Immunogen
Source: mBio. 2021 Jul 6;12(4):e02531-20. doi: 10.1128/mBio.02531-20 (PMC8406324; doi:10.1128/mBio.02531-20)
Supplement: TABLE S3 [file mbio.02531-20-st003.docx]

| **Virus** | **Protein** | **Site** | **mAb evaded** | **Homologous position in HTNV Gn** | **Reference** |
| --- | --- | --- | --- | --- | --- |
| HTNV | Gn | K76E | mAb 3D5 and mAb 16D2 | K76 | Kikuchi et al |
| HTNV | Gn | G217R | mAb 2D5 | G217 | Wang et al |
| HTNV | Gn | P303T | mAb 16E6 | P303 | Wang et al |
| HTNV | Gn | H304Y | mAb 3D5 | H304 | Wang et al |
| PUUV | Gn | D272V | mAb 5A2 | D262 | Horling et al |
| ANDV | Gn | N108K | mAb KL-AN-4E1 | D104 | Duehr et al |
| ANDV | Gn | D121V | mAb KL-AN-3F6 | E117 | Duehr et al |
| ANDV | Gn | K124N | mAb KL-AN-4G11 | Y120 | Duehr et al |
| ANDV | Gn | K225R | mAb KL-AN-4H6 | K221 | Duehr et al |
