# Supplementary material for: Structural Basis for a Neutralizing Antibody Response Elicited by a Recombinant Hantaan Virus Gn Immunogen
Source: mBio. 2021 Jul 6;12(4):e02531-20. doi: 10.1128/mBio.02531-20 (PMC8406324; doi:10.1128/mBio.02531-20)
Supplement: TABLE S4 [file mbio.02531-20-st004.docx]

|  |
| --- |

|  | **HTNV VLP** | **HTNV VLP + HTN-Gn1**^b^ |
| --- | --- | --- |
| Tilt range (degrees) | –30–60 | –42–60 |
| Tilt increment (degrees) | 3 | 3 |
| Frames per tilt | 6 | 8 |
| Electron exposure (e⁻/ Å²/tilt) | 4.50 | 4.77 |
| Total electron exposure (e⁻/ Å²) | 139.5 | 167.0 |
| Pixel size (Å) | 1.36 | 1.76 |
| Defocus range (μm) | 2.8–4.0 | 2.8–4.0 |
| Tilt series | 85 | 43 |
| Tomograms | 69 | 42 |
| VLP sub-volumes | 95 | 55 |
| EMDB ID | EMD-12543 | EMD-12544 |
| Spike sub-volumes | 3,209 | 2,380 |
| Symmetry | C4 | C1 |
| Pixel size (Å) | 2.72 | 7.04 |
| Resolution (Å) | 12.3 | 19.0 |
| Model-to-Map CC^a^ | n/a | 0.9 |
| ^a^CC: Correlation coefficient from Chimera ‘Fit in map’ function  ^b^Fitted PDB: 7NRH | | |
|  | | |
